# Long-Tailed Macaque Response to Deforestation in a *Plasmodium knowlesi-*Endemic Area

**DOI:** 10.1007/s10393-019-01403-9

**Published:** 2019-03-29

**Authors:** Danica J. Stark, Kimberly M. Fornace, Patrick M. Brock, Tommy Rowel Abidin, Lauren Gilhooly, Cyrlen Jalius, Benoit Goossens, Chris J. Drakeley, Milena Salgado-Lynn

**Affiliations:** 1 Organisms and Environment Division, Cardiff School of Biosciences, Cardiff University, Sir Martin Evans Building, Museum Avenue, Cardiff, CF10 3AX UK; 2 Danau Girang Field Centre, c/o Sabah Wildlife Department, Wisma Muis Block B Floor 5, 88100 Kota Kinabalu, Sabah Malaysia; 3 Faculty of Infectious and Tropical Diseases, London School of Hygiene and Tropical Medicine, London, UK; 4 Institute of Biodiversity, Animal Health and Comparative Medicine, College of Medical, Veterinary and Life Sciences, University of Glasgow, Glasgow, G61 1QH UK; 5 Infectious Diseases Society Kota Kinabalu- Menzies School of Health Research Clinical Research Unit, Kota Kinabalu, Malaysia; 6 Department of Anthropology, Faculty of Social Sciences, University of Western Ontario, Social Science Building, London, N6A 3K7 Canada; 7 Wildlife Health, Genetic and Forensic Laboratory, Sabah Wildlife Department, Wisma Muis, 88100 Kota Kinabalu, Sabah Malaysia; 8 Sabah Wildlife Department, Wisma Muis, 88100 Kota Kinabalu, Sabah Malaysia; 9 Sustainable Places Research Institute, Cardiff University, 33 Park Place, Cardiff, CF10 3BA UK

**Keywords:** Deforestation, Remote sensing, Habitat selection, Malaria, Borneo, GPS collar

## Abstract

Land-use changes can impact infectious disease transmission by increasing spatial overlap between people and wildlife disease reservoirs. In Malaysian Borneo, increases in human infections by the zoonotic malaria *Plasmodium knowlesi* are hypothesised to be due to increasing contact between people and macaques due to deforestation. To explore how macaque responses to environmental change impact disease risks, we analysed movement of a GPS-collared long-tailed macaque in a *knowlesi*-endemic area in Sabah, Malaysia, during a deforestation event. Land-cover maps were derived from satellite-based and aerial remote sensing data and models of macaque occurrence were developed to evaluate how macaque habitat use was influenced by land-use change. During deforestation, changes were observed in macaque troop home range size, movement speeds and use of different habitat types. Results of models were consistent with the hypothesis that macaque ranging behaviour is disturbed by deforestation events but begins to equilibrate after seeking and occupying a new habitat, potentially impacting human disease risks. Further research is required to explore how these changes in macaque movement affect *knowlesi* epidemiology on a wider spatial scale.

**Electronic supplementary material:**

The online version of this article (10.1007/s10393-019-01403-9) contains supplementary material, which is available to authorized users.

## Introduction

Deforestation and associated habitat disturbance have been associated with changing infectious disease transmission by bringing wildlife disease reservoirs into closer proximity to people (Patz et al. 2004). This response to habitat loss is particularly relevant for non-human primates, as an estimated 90% of all primate species live in tropical areas and depend on forests that are rapidly disappearing (Chapman and Peres 2001). Within Malaysian Borneo, the increased detection of the zoonotic malaria *Plasmodium knowlesi* (henceforth *knowlesi*) in humans is hypothesised to be driven by increasing spatial overlap between human populations and simian hosts (Imai et al. 2014).

Large numbers of human *knowlesi* infections were only identified in 2004 in Malaysian Borneo (Singh et al. 2004). Since then, human *knowlesi* infections have been identified throughout Southeast Asia and *knowlesi* is now the main cause of clinical malaria in Malaysia (William et al. 2013; Shearer et al. 2016). Changes in the numbers and proportions of suspected *knowlesi* case notifications within the Malaysian state of Sabah suggest that incidence of the disease is increasing (William et al. 2013). In Sarawak, Malaysia, *knowlesi* has been found in wild long-tailed and pig-tailed macaques (*Macaca fascicularis* and *M. nemestrina*) (Lee et al. 2011); however, genetic studies of macaque and human parasites suggest *knowlesi* remains primarily a zoonosis, with macaques acting as reservoir hosts (Lee et al. 2011; Divis et al. 2015).

Land-use changes are believed to be the primary driver of this apparent emergence. In Northern Sabah, Malaysia, an area undergoing rapid environmental change through conversion of forest to agricultural land, deforestation has been associated with increases in reported incidence of *knowlesi*; however, the mechanisms underlying this association remain unknown (Fornace et al. 2016). While changes in forest cover affect the abundance of mosquito vectors and new settlements can increase the number of people at risk, a key determinant of risk is the proximity and infection status of macaque populations to susceptible individuals (Imai et al. 2014). Within this area of Sabah, a recent case control study found individuals reporting proximity to macaques in the previous month had over three times the odds of *knowlesi* infection (Grigg et al. 2017).

As the geographical range of long-tailed macaques is one of the most widespread ranges of non-human primates (Fooden 1995), and due to the rapid rate at which forest is being cleared within their natural range in Southeast Asia, many long-tailed macaque populations are now largely ecologically associated with humans (Sha et al. 2009; Fuentes 2011; Moyes et al. 2016). Although long-tailed macaques are flexible in their feeding strategies, and able to adapt to changing environments (Menard 2004; Gumert 2011), how they adjust their ranging patterns during periods of disturbance in their home range is not well understood. Primates are generally known to be able to create a cognitive map that allows them to travel efficiently between food patches (Janson and Byrne 2007). Whether their home ranges are within forests or urban areas, their familiarity within the area results in relatively stable home ranges (Milton and May 1976). However, when the habitat within their spatial memory map is changed, the ability to adjust and/or re-establish a new cognitive map is important for such a species in a changing environment.

To explore how macaque’s response to environmental changes may contribute to human disease risks, we analysed movement data of a long-tailed macaque that was GPS-collared prior to a deforestation event in close proximity to a human settlement in a *knowlesi-*endemic area of Northern Sabah. We aimed to (1) examine how ranging patterns changed throughout the deforestation event and (2) evaluate how the predictability of macaque habitat selection was influenced by the deforestation and disturbance.

## Methods

### Study Site

**Figure 1:**
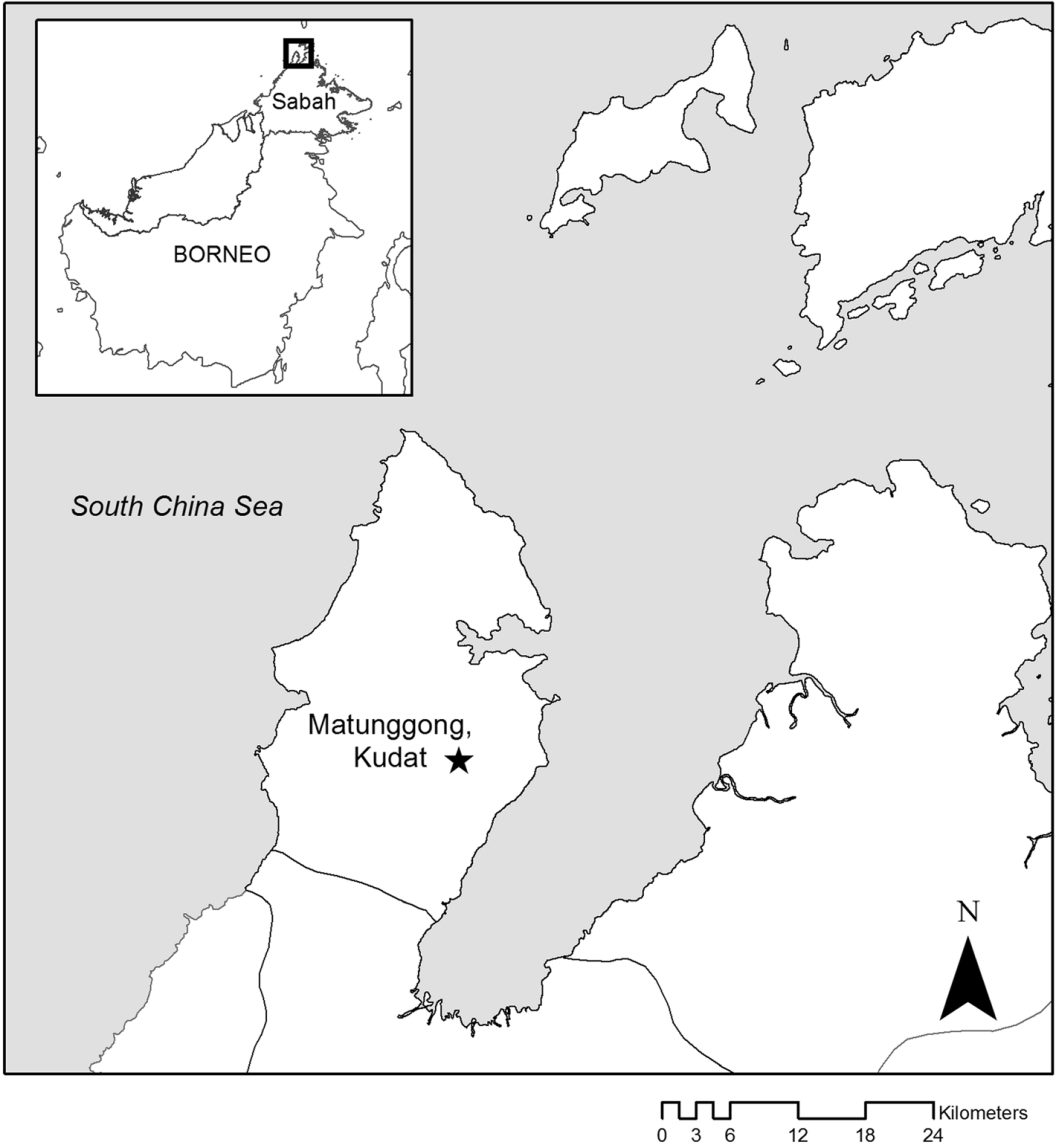
Map showing study site in Kudat, Sabah, Malaysian Borneo. Star indicates where the long-tailed macaque was collared and the clearing occurred during the study period.

**Figure 2:**
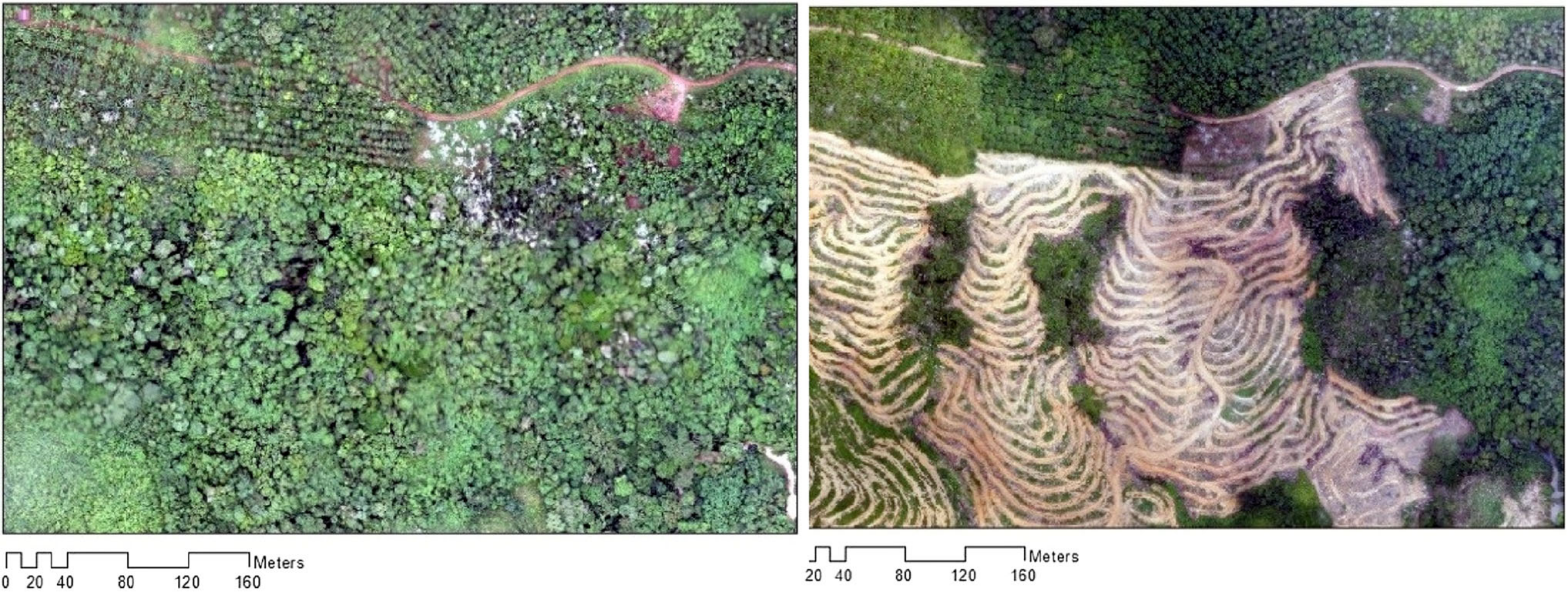
Examples of aerial imagery collected by UAV pre- and post-deforestation within the study area.

This study was conducted in a largely deforested and highly fragmented case study area in Kudat district in Sabah, Malaysia (N6.77, E116.78, 10–150 m above sea level, Fig. 1). The site was selected as a representative area where *knowlesi* transmission is occurring based on household locations of symptomatic cases recruited at district hospitals (Grigg et al. 2017) and is the focus of a number of linked interdisciplinary studies on *knowlesi* ecology, including entomological, sociological and epidemiological studies (http://malaria.lshtm.ac.uk/MONKEYBAR). This area includes 11 villages with a total population of 1160 individuals and had a *knowlesi* incidence of 12 cases per 1000 individuals in 2014 (Fornace et al. in press). High densities of *Anopheles balabacensis* have been reported in settled and agricultural lands in this site, and mosquitoes infected by *knowlesi* and other simian malarias have been identified (Wong et al. 2015; Manin et al. 2016). This is a low elevation, coastal area dominated by small-scale agriculture, including swidden farming and small rubber and oil palm plantations. There are high rates of land-use change and, during the study period, large-scale forest clearing for a rubber plantation. Land-cover maps were derived from Landsat and aerial imagery for six periods corresponding to stages of deforestation that occurred during this study (Fig. 2). The deforestation event refers to the entire study period whereby forest loss was being observed. The deforestation event was divided into six periods, which included a pre-clearing (Period 1) and post-clearing (Period 6) stage. Although there may have been some clearing going on during the pre- and post-clearing periods, it was outside of our main study area and therefore not considered. The periods of active land clearing were divided based on a combination of the imagery available from the Landsat and aerial flights and ensuring there were sufficient numbers of GPS fixes for home range estimation (Benhamou and Cornelis 2010).

### Macaque Collaring and Sampling

An adult male long-tailed macaque was caught in a baited trap and was sedated by an authorised veterinarian in February 2014. A VHF/GPS remote-download, automatic self-releasing collar (Telemetry Solutions Quantum 4000E) was fitted, which weighed ~ 2.5% of the individual’s body mass [cf. recommended 5% maximum (American Society of Mammalogists 1998)]. The collar was pre-programmed to record data points every 30 min from 05:00 to 07:00 and 17:00 to 19:00, and every other hour between these times of the day, automatically releasing after 169 days. While the animal was sedated, 2 ml of blood was collected in an EDTA-containing tube and kept refrigerated until processing (Supplementary Material).

At the time of collaring, the macaque belonged to an unhabituated group that typically fled upon seeing humans. No effort was made to habituate the group in order to ensure that the home range data were not affected by researcher presence. Group size was therefore estimated using auditory cues from macaque movement in the trees since visual sightings were rare. The movement of one individual is often used as a proxy for primate troop movements (e.g. Ren et al. 2009; McLean et al. 2016; Stark et al. 2017), and this assumption was validated by visual and auditory cues during field surveys; therefore, the locations of the collared individual were considered representative of the group as a whole.

### Home Range Estimation

To calculate home range estimates and examine shifts in range and movement rates over the different clearing periods, biased random bridges (BRB) were used (Benhamou 2011; Stark et al. 2017). The maximum time threshold (*T*_max_), the longest period between points before they are no longer autocorrelated, was set to 7200 s. Based on the average distances between fixes during static collar tests, the length threshold of movement (*L*_min_) was set to 15 m. This assumed that tracks less than *L*_min_ were assumed resting or due to GPS error. The minimum smoothing parameter (*H*_min_) was set at 25 m, which was large enough to encompass all potential locations the animal could occur within while being recorded at the same location, while not being larger than half the mean distance travelled for the time *T*_max_. Diffusion rates were estimated for each land-use type using the land-cover map. The utilisation distributions (UDs) were based on the 90th percentile for overall home range size and 50th percentile for the core area.

### Habitat Use Modelling

To test whether macaque habitat use within a 1000 m range was influenced by the deforestation event, binomial generalised additive models (GAMs) were fitted to eight habitat variables for each of the six time periods. Habitat variables were: aspect, slope, elevation, distance to nearest road, distance to nearest house, distance to nearest forest, distance to nearest agriculture and distance to nearest clearing (at 30 m^2^ resolution). For each period, the same number of absence points as there were presence points were randomly sampled from an area within 1000 m from all included presence points. Absence points were re-sampled 100 times for each period, and models run on each of these data sets.

To explore whether macaque habitat use was influenced by the disturbance of deforestation during periods of active land clearing, binomial generalised additive models fitted as above to periods 3, 4 and 5, but with an additional variable: distance to recent deforestation (i.e. between periods 2 and 3, 3 and 4, and 4 and 5, respectively). All statistical analyses and land-cover classification were conducted in R statistical software (R Foundation for Statistical Computing, Vienna, Austria). Maps were visualised using ArcGIS (ESRI, Redlands, USA).

## Results

### Macaque Troop Size and Knowlesi Infection

The collared animal was found PCR positive for *Plasmodium* spp., specifically *knowlesi*. Obtaining an accurate group size estimate both pre- and post-clearing was difficult due to the skittish and elusive nature of this unhabituated macaque group, but was estimated at ≥ 40 individuals near the time of collaring, and fell to ~ 20 individuals following the deforestation, suggesting a group fission event may have occurred.

### Land-Cover Changes and Macaque Ranging Behaviours

**Figure 3:**
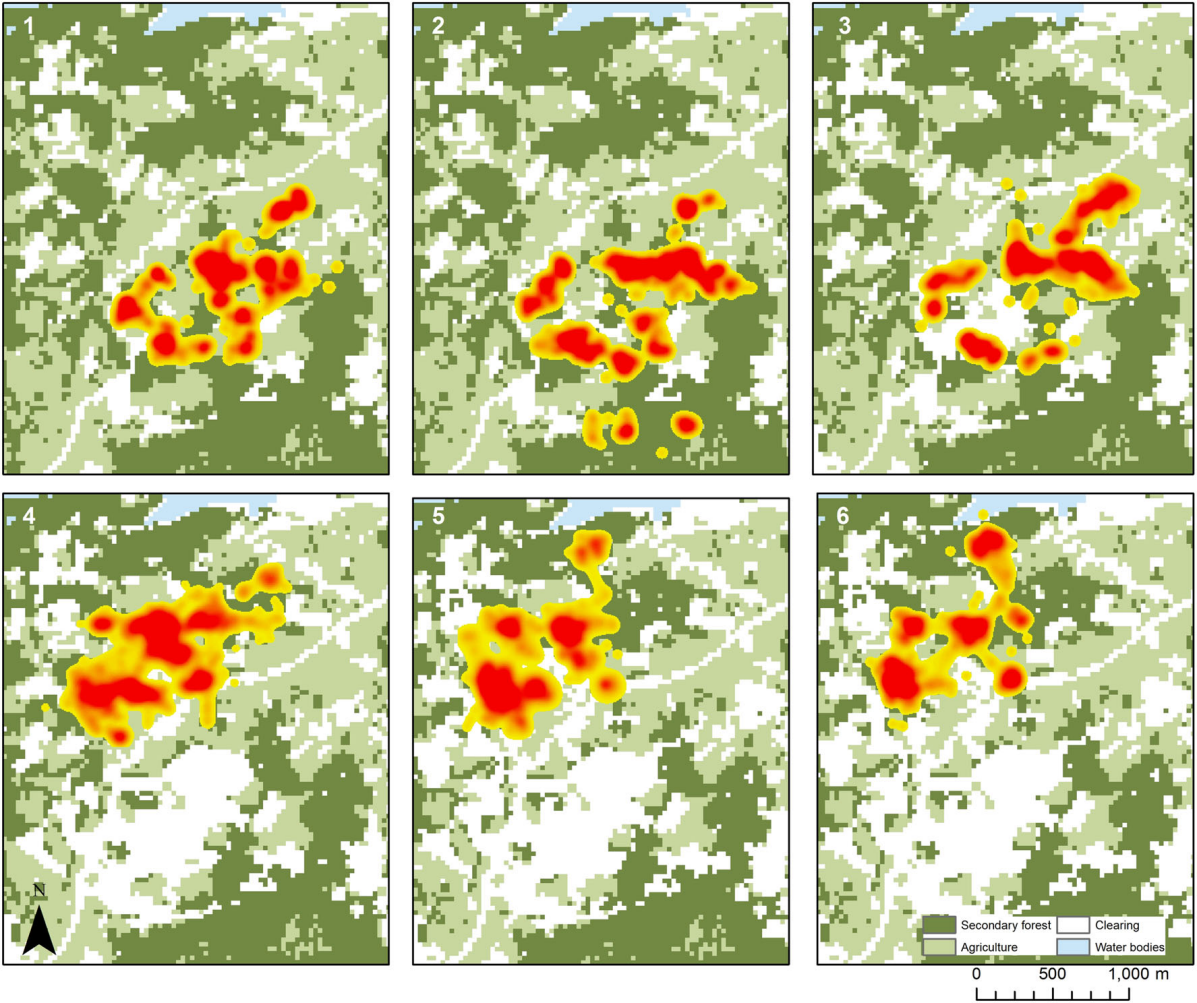
Utilisation distributions and estimated home ranges (ha) for the collared macaque. Time periods for clearing events: (1) February 2014, (2) March–April 2014, (3) April–May 2014, (4) May–June 2014, (5) June–August 2014 and (6) August–October 2014.

Between February and September 2014, a total of 190 ha were cleared within a 1000 m buffer area around the macaque troop home range (Table 1). This included a loss of 95.5 ha of secondary forest, representing 14% of the available secondary forest within this home range area. The home range and core range of the collared individual declined in area as the clearing progressed, with the core area decreasing from 10.3 ha prior to the clearing to less than 6 ha after the clearing was completed (Fig. 3). The proportion of time the macaque spent in forested areas vs other habitats also changed as clearing progressed, with the ratio of points in the forest to non-forest dropping during peak clearing periods (pre-clearing: 2.69, peak clearing: 0.93 and up to 6.38 post-clearing). There was an increase in fixes in farmland/agricultural lands during the peak clearing periods, from 25% of the points in Period 1 to 41% in Period 3, and then dropped down to 9% after the major clearing had finished (Period 6). Diffusion rates further suggest that the macaque moved through non-forest areas faster during the peak periods of forest clearing (Periods 3, 4 and 5).

**Table 1:** Land-Use Change and Macaque Ranging Characteristics.

	Period 1 Before clearing	Period 2 March–April 2014	Period 3 May–June 2014	Period 4 June–July 2014	Period 5 July–Aug 2014	Period 6 After clearing
Land-use characteristics						
Forest area (ha)	669.5	664.6	646.9	610.5	574.0	574.0
Core forest area excluding edges (ha)	335.6	328.5	315.4	294.1	273.8	273.8
Cleared/open areas (ha)	192.9	201.2	248.2	318.1	382.5	382.5
Net forest loss (ha)	–	5.0	22.6	59.0	95.5	95.5
Cumulative proportion area cleared (%)	–	0.6%	4.0%	9.1%	13.7%	13.7%
Macaque ranging						
Home range size (ha)	38.53	44.69	36.38	44.31	36.50	25.85
Core range size (ha)	10.29	11.98	9.69	9.97	8.24	5.67
Distance from houses (median—m)	310.3	284.5	248.8	285.0	346.7	421.6
Diffusion rate (global) (m^2^/s)	1.52	1.13	0.97	2.78	2.46	0.81
Forest	1.08	0.60	0.55	0.52	0.65	0.35
Farmland	0.14	0.15	0.58	0.30	–	–
Clearing	–	–	0.18	–	–	–

### Macaque Habitat Use

**Figure 4:**
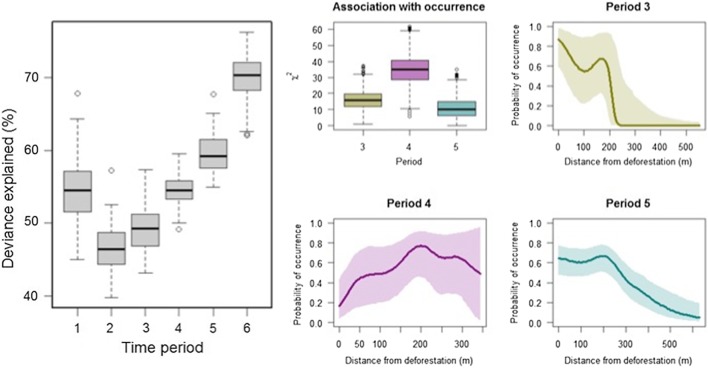
Deviance explained by 100 binomial generalised additive models fit for each time period (left), and associations between the probability of occurrence and distance to recent deforestation for peak clearing periods (right).

Binomial generalised additive models fit separately across periods suggest varying associations between macaque occurrence and habitat characteristics. The median proportion of deviance explained by 100 models fit for each period decreases as clearing begins and then increases following the main clearing activities (Fig. 4). When distance from recent deforestation was included in models during peak clearing periods, the strongest association between distance from deforestation and macaque occurrence occurred during Period 4 (i.e. in response to forest loss between Periods 3 and 4), with every model suggesting a strong effect, and the fitted curve suggesting avoidance relative to other periods (Fig. 4). The next strongest association occurred during Period 3, with approximately 80% of models suggesting a significant association, and the association was weakest during Period 5, with only 50% of models suggesting a significant association.

## Discussion

Although these data only represent a single macaque troop, results are consistent with the hypothesis that macaque ranging behaviour is disturbed by deforestation events, but begins to equilibrate after the troop seeks a new suitable habitat and moves to occupy it. During a large-scale clearing over a period of several months, changes were observed in the macaque troop group size, home range size, movement speeds and use of different habitat types. Statistical evidence indicates deforestation influenced the behaviour of macaques for this troop, which could be important in understanding how deforestation impacts *knowlesi* transmission.

Changes in group size may be due to a fluid fission–fusion social structure, especially in response to resource stress and habitat disturbance reported in some primate species [e.g. *Ateles geoffroyi* (Rodrigues 2017); *Varecia variegata* (Holmes et al. 2016)], although long-term data on this are scarce. After a severe forest fires in Kalimantan, macaques adjusted to low fruit and flower availability by relying on fallback foods (e.g. insects), by foraging in a more dispersed group, and by increasing the amount of time spent travelling on the ground (Berenstain 1986). It seems likely that the macaques in the current study responded to a drastic reduction in resource availability by splitting into two or more groups. Further long-term monitoring of macaques after complete deforestation events is necessary to determine whether such changes in group size are permanent.

While these models cannot be used to extrapolate to macaque distribution and habitat selection broadly, within this range, macaque occurrence during periods of active deforestation was associated with distance from recent clearing, suggesting avoidance of logging activities. Similar changes to home range and behaviour have been reported in other primate species in response to selective logging (e.g. Johns 1986) and initial decreases in *M. fascicularis* populations have been reported from selectively logged areas in Malaysian Borneo (Brodie et al. 2014). Variations in deviance explained by habitat models for each period suggest movements become less predictable during times of active clearing before returning closer to equilibrium once deforestation activities are completed (Fig. 4). Changes to macaque habitat and home range may have implications for infectious disease transmission, for example by bringing primates into closer proximity to people (e.g. Chapman et al. 2006; current study), or increasing population density in smaller forest fragments leading to higher contact rates between primates (e.g. Chapman et al. 2005). These anthropogenic land-use changes may additionally alter ecological community composition, vector breeding sites or human use of different environments, leading to further changes in disease risks (Young et al. 2013; Lambin et al. 2010). Furthermore, an increase in physiological and psychological stress can lead to macaques becoming immunocompromised, and thereby increasing macaque morbidity and mortality (Nunn and Altizer 2006).

Blood taken from the macaque at the time of collaring was positive for *knowlesi*. Although samples were not collected from the same animal in subsequent occasions nor from other members of the same troop, estimates of troop-level *Plasmodium* prevalence within other troops in the Kudat district were 87% (CI 67–98%) (MSL, unpublished data). During the period of deforestation, 15 human cases of *knowlesi* were reported within 3 km of the macaque roosting sites, with the highest number of cases reported in June through July (Grigg et al. 2017). All cases were reported in adults (age range 19–59), and the majority were male (12/15). While it is impossible to determine whether these cases were related to changes in macaque movement, these observations are consistent with the possibility that the spike in human cases arose due to a knock-on effect of deforestation and therefore merits further investigation.

## Conclusion

Despite efforts to collar additional troops in the area, attempts to collar additional macaques in this agriculture–forest matrix were unsuccessful and may itself be an example of how land development impacts the distribution and behaviour of primates. However, these results indicate changes to macaque behaviour and space use in response to anthropogenic environmental change. Future work should include increasing the number of groups or individuals collared, in order to better understand the macaques’ response to habitat clearing. However, it is difficult to obtain these data (including pre-, peak- and post-clearing movement data), as forest clearing plans are not always made known to others or done legally. These findings have important implications for understanding the epidemiology of *knowlesi* and other zoonotic diseases carried by long-tailed macaques (e.g. helminth infections, simian foamy virus and herpes B) and should be explored further through more extensive studies or mathematical modelling approaches.

## Electronic supplementary material

Below is the link to the electronic supplementary material.

**Supplementary Material:** Detailed methodology for land classification and the molecular diagnosis of *Plasmodium spp.* and *P. knowlesi* (DOCX 19 kb)
