# Supplementary material for: Long-Tailed Macaque Response to Deforestation in a Plasmodium knowlesi-Endemic Area
Source: Ecohealth. 2019 Mar 29;16(4):638–46. doi: 10.1007/s10393-019-01403-9 (PMC6910895; doi:10.1007/s10393-019-01403-9)
Supplement: Supplementary file 1 — Supplementary Material: Detailed methodology for land classification and the molecular diagnosis of Plasmodium spp. and P. knowlesi (DOCX 19 kb) [file 10393_2019_1403_MOESM1_ESM.docx]

**Supplementary Material:**

**Long-Tailed Macaque Response to Deforestation in a *Plasmodium knowlesi*-Endemic Area**

*Land Classification*

During the study period, the Sensefly eBee Unmanned Aerial Vehicle (UAV or drone; Sensely, Cheseaux-Lausanne, Switzerland) was used to repeatedly map areas of the study site, as described in (Fornace et al. 2014). Flights were programmed and monitored using eMotion software (Sensefly, Chesaux-Lausanne, Switzerland) and post-flight processing was completed using Postflight Terra 3D (Pix4D SA, Lausanne, Switzerland). Airborne data collection was performed at 400 m above ground level, generating high-resolution data with an average of 11cm per pixel. Aerial images and digital surface models were produced for areas of the study site; these were manually digitised and classified into four land use types (secondary forest, agricultural land, cleared or open areas and water bodies) using local knowledge and field validation.

As the aerial images did not cover the full extent of the macaque home range, these data were used to develop training files for classification of satellite-based remote sensing data. Landsat 8 images at 30m resolution and digital elevation data from the ASTER Global Digital Elevation Model were utilised (Land Processes Distributed Active Archive Center (LP DAAC) 2014, 2015). Normalised vegetation indices were also calculated from Landsat satellite data. Ground surveys using Global Positioning Systems (GPS) were used to map locations of all houses and roads; these were used to create raster surfaces of distances from roads. All data was resampled to 30 m resolution. Due to high levels of cloud cover in this area from July – September 2014, data from following months was used and validated using aerial UAV images collected during August 2014.

A random forest classifier was used to classify six images, corresponding to different stages of clearing. This algorithm is an ensemble classifier which uses multiple decision trees to predict a test set based on training data (Breiman 2001; Gislason, Benediktsson, and Sveinsson 2006). Each tree was grown with different bootstrapped samples of two-thirds of the training data, with the remaining third of the data used to derive an “out-of-bag” (OOB) error. All random forests were run using 10,000 trees to ensure stability. Random forest models were run iteratively, with least predictive variables excluded at every run to obtain the lowest OOB as described by (Diaz-Uriarte 2007). To independently validate the classification, 100 data points were withheld from the training data and compared to the predicted classification. The land classification was highly accurate (Kappa accuracy > 95%), although some areas of secondary forest were indistinguishable from tree plantations.

*Molecular Diagnosis of Plasmodium spp. and P. knowlesi*

Total genomic DNA was extracted from the long-tailed macaque blood sample using a DNeasy Blood and Tissue Kit (QIAGEN®) following the manufacturer’s recommendations for “purification of total DNA from animal blood or cells” (spin-column protocol). Nested PCRs for *Plasmodium* spp. detection was performed following the method of Siregar *et al.* targeting the mitochondrial small subunit ribosomal RNA (ssrRNA) (Siregar et al. 2015). A 25 µl total volume of PCR mix consisted of 1X Phusion HF Buffer (New England Biolabs, Thermo Scientific ^TM^), 0.02 U/µl of Phusion High Fidelity Polymerase (New England Biolabs, Thermo Scientific ^TM^), 0.4 µM of each primer (PFf4595 and PFr5019), 0.1mM (each) dNTPs (Promega), and ̴0.3 ng/µl of DNA template for the Nest 1 reaction and 1µl of the Nest 1 PCR product for the Nest 2 reaction mix. PCRs were performed in a Veriti Thermal Cycler (Applied Biosystems) under the following profile for both nests: 94°C for 5 min, 30 cycles at 94°C for 15 s, 60°C for 15 s, 72°C for 45 s and a final extension at 72°C for 5 min. The PCR products were electrophoresed in a 1.5% agarose gel and results were accepted as positives if the presence of band of ̴424 bp was detected.

Molecular detection of *P. knowlesi* was performed following the method of Imwong *et al.* also targeting the mitochondrial ssrRNA (Imwong et al. 2009). A 10 µl total volume of PCR mix consisted of 1X Multiplex Plus Kit (QIAGEN), 0.25 µM of each primer (PlU1 and PkR1150 for Nest 1; PkF1140 and PkR1150 for Nest 2), and ̴4 ng/µl of DNA template for the Nest 1 reaction and 2µl of the Nest 1 PCR product for the Nest 2 reaction mix (Singh et al. 1999). PCRs were performed under the following profiles: 1) Nest 1, 95°C for 5 min, 30 cycles at 55°C for 1 min, 72°C for 1 min, 94°C for 1min, then a final annealing and extension at 55°C and 72°C for 1 min each; 2) Nest 2, 95°C for 5 min, 30 cycles at 50°C for 1 min, 72°C for 1 min, 94°C for 1min, then a final annealing and extension at 50°C and 72°C for 1 min each. Electrophoresis was performed as above and positive results were considered as the presence of a ̴450 bp band.

Breiman, L. 2001. 'Random Forests', *Machine Learning*, 45: 5-32.

Diaz-Uriarte, R. 2007. 'GeneSrF and varSelRF: a web-based tool and R package for gene selection and classification using random forest', *BMC Bioinformatics*, 8: 328.

Fornace, K. M., C. J. Drakeley, T. William, F. Espino, and J. Cox. 2014. 'Mapping infectious disease landscapes: unmanned aerial vehicles and epidemiology', *Trends Parasitol*, 30: 514-19.

Gislason, P.O., J.A. Benediktsson, and J.R. Sveinsson. 2006. 'Random forests for land cover classification', *Pattern Recognition Letters*, 27: 294-300.

Imwong, M., N. Tanomsing, S. Pukrittayakamee, N. P. Day, N. J. White, and G. Snounou. 2009. 'Spurious amplification of a Plasmodium vivax small-subunit RNA gene by use of primers currently used to detect P. knowlesi', *J Clin Microbiol*, 47: 4173-5.

Land Processes Distributed Active Archive Center (LP DAAC). 2014. "Landsat 8 Operational Land Imager." In. Sioux Falls, South Dakota: NASA EOSDIS Land Processes DAAC, USGS Earth Resources Observation and Science (EROS) Center.

———. 2015. "Advanced Spaceborne Thermal Emission and Reflection Radiometer Global Digital Elevation Model (ASTER GDEM) Version 2." In. Sioux Falls, South Dakota: NASA EOSDIS Land Processes DAAC, USGS Earth Resources Observatoin and Science (EROS) Center.

Singh, B., A. Bobogare, J. Cox-Singh, G. Snounou, M. S. Abdullah, and H. A. Rahman. 1999. 'A genus- and species-specific nested polymerase chain reaction malaria detection assay for epidemiologic studies', *Am J Trop Med Hyg*, 60: 687-92.

Siregar, J. E., C. L. Faust, L. S. Murdiyarso, L. Rosmanah, U. Saepuloh, A. P. Dobson, and D. Iskandriati. 2015. 'Non-invasive surveillance for Plasmodium in reservoir macaque species', *Malar J*, 14: 404.
